# Assessment of the structure of the Hospital Anxiety and Depression Scale in musculoskeletal patients

**DOI:** 10.1186/1477-7525-3-82

**Published:** 2005-12-19

**Authors:** Julie F Pallant, Catherine M Bailey

**Affiliations:** 1 Faculty of Life and Social Sciences, Swinburne University of Technology, P.O. Box 218, Hawthorn, Victoria 3122, Melbourne, Australia

## Abstract

**Background:**

Research suggests there is a high prevalence of anxiety and depression amongst patients with chronic musculoskeletal pain, which can influence the effectiveness of rehabilitation programs. It is therefore important for clinicians involved in musculoskeletal rehabilitation programs to consider screening patients for elevated levels of anxiety and depression and to provide appropriate counselling or treatment where necessary. The HADS has been used as a screening tool for assessment of anxiety and depression in a wide variety of clinical groups. Recent research however has questioned its suitability for use with some patient groups due to problems with dimensionality and the behaviour of individual items. The aim of this study is to assess the underlying structure and psychometric properties of the HADS among patients attending musculoskeletal rehabilitation.

**Methods:**

Data was obtained from 296 patients attending an outpatient musculoskeletal pain clinic. The total sample was used to identify the proportion of patients with elevated levels of anxiety and depression. Half the sample (n = 142) was used for exploratory factor analysis (EFA), with the holdout sample (n = 154) used for confirmatory factor analysis (CFA) to explore the underlying structure of the scale.

**Results:**

A substantial proportion of patients were classified as probable cases on the HADS Anxiety subscale (38.2%) and HADS Depression subscale (30.1%), with the sample recording higher mean HADS subscales scores than many other patient groups (breast cancer, end-stage renal disease, heart disease) reported in the literature. EFA supported a two factor structure (representing anxiety and depression) as proposed by the scale's authors, however item 7 (an anxiety item) failed to load appropriately. Removing Item 7 resulted in a clear two factor solution in both EFA and CFA.

**Conclusion:**

The high levels of anxiety and depression detected in this sample suggests that screening for psychological comorbidity is important in musculoskeletal rehabilitation settings. It is necessary for clinicians who are considering using the HADS as a screening tool to first assess its suitability with their particular patient group. Although EFA and CFA supported the presence of two subscales representing anxiety and depression, the results with this musculoskeletal sample suggest that item 7 should be removed from the anxiety subscale.

## Background

Anxiety and depression are major factors impacting patient's quality of life, and the associated symptoms (inability to concentrate, loss of motivation, disturbed sleep, fatigue, pessimistic mood) may influence their ability to benefit from treatment and rehabilitation programs. High levels of anxiety have been associated with poor concentration and difficulty in comprehending information provided by clinicians [1]. Depressed mood may also adversely affect patients' willingness to comply with prescribed medications and to undertake the necessary lifestyle changes (eg. exercise, diet).

Studies have found quite high levels of psychological distress among patients with musculoskeletal diseases in particular [2,3]. It is therefore important for clinicians involved in musculoskeletal rehabilitation programs to consider screening patients for elevated levels of anxiety and depression and to provide appropriate counselling or treatment where necessary. Reduction in levels of anxiety and depression should also be considered an important program outcome.

One of the issues facing clinicians wishing to screen for high levels of anxiety and depression among their patients is the choice of a screening tool. In a hospital setting, the tool needs to be quickly administered, easy to use and have good psychometric properties. Many of the available measures (eg. Beck Depression Inventory [4]) are quite long and detailed, and are restricted for use by psychologists, psychiatrists, or other suitably trained personnel. This makes them unsuitable for routine administration as part of the normal pre-program assessment procedures.

In the selection of assessment tools for screening and evaluation there are a number of issues to be considered. Guyatt, Feeny, and Patrick [5] in their guidelines on the selection of health related quality of life measures highlight a number of factors that are relevant here (for a more comprehensive review see Streiner and Norman, [6]). Measures need to be reliable (internally consistent, and stable over time), valid (measuring the intended characteristic), and responsive (able to detect change). Reliability, accuracy and reproducibility are important qualities for a discriminative instrument (for use as a screening tool in distinguishing those with high versus low levels of a characteristic). For an evaluative instrument, responsiveness (as indicated by sensitivity to detect changes in patients who have improved or deteriorated) is also essential.

One of the tools that could be considered for use as a discriminative measure in the rehabilitation context is the Hospital Anxiety and Depression Scale (HADS) [7]. It was originally developed as a short questionnaire designed to identify clinical 'caseness' for anxiety and depression in general medical outpatient populations. The items included in the HADS were chosen to reduce contamination with somatic symptoms, which are common in patient populations. The HADS consists of an anxiety and depression subscale, each containing seven items, making it quick and easy to use in clinical settings.

Since its publication in the early 1980's the HADS has been used in a growing number of studies across a variety of patient groups and clinical contexts (see review by Bjelland, et al., [8]). As a brief screening tool the HADS scale is increasing in popularity, particularly in clinical settings, because of its ease of administration and independence from physical symptomatology. After reviewing over 70 journal articles Bjelland and coauthors [8] concluded that the HADS 'performs well in screening for the separate dimensions of anxiety and depression and caseness of anxiety disorders and depression in patients from non-psychiatric hospital clinics' (p. 75).

The HADS was originally designed to assess two separate dimensions of anxiety and depression. The case for the bidimensionality of the HADS was supported in a review paper published by Bjelland et al. [8]. Of the 18 studies reviewed that reported findings of factor analyses, eleven of the papers supported a two-factor solution. A strong argument for the use of the two factors is also made at the clinical level [9] where it is clinically relevant to separately determine levels of anxiety and depression.

In twenty-one studies investigating the HADS, the Pearson correlation coefficients between the two subscales of anxiety and depression were reported to have a mean of .56 [8]. These high correlation rates have encouraged some authors to question the dimensionality of the scale. Razavi, Delvaux, Farvacques and Robaye [10] recommended totalling the two subscales to create a total score of generalised psychological distress. Martin, Tweed and Metcalfe [11] in a study of patients with end-stage renal disease, suggested that it may be 'appropriate to consider adopting a global total score of psychological distress' (p. 61) as an alternative to the original two subscale structure.

A growing number of authors have argued that there are three or more factors contained within the scale [12-14]. In a recent review of the HADS, Martin [15] states that 'there is accumulating evidence that the fundamental factor structure of the HADS comprises not two, but three factors and indeed, that the three-factor structure offers a superior fit to clinical data than the two factor (anxiety and depression model)' (p.70). Drawing on Clark and Watson's [16] tripartite theory of anxiety and depression, Dunbar and colleagues [12] found empirical support using confirmatory factor analysis (CFA) for a three factor model of the HADS, distinguishing autonomic anxiety (items 3,9,13), negative affectivity (items 1,5,7,11) and anhedonic depression (items 7,2,4,6,8,10,12,14). Item 7 (an anxiety item) was found to load on two factors (negative affectivity and depression). Martin [15] suggests that the autonomic arousal component of the HADS anxiety subscale may be a potential confounding factor when used with patients experiencing physical symptoms, affecting its reliability as a screening tool.

Another issue that has been raised in regard to the HADS is the appropriateness of individual items with specific patient groups [17]. The review by Bjelland et al. [8] revealed that the anxiety item 7 "*I can sit at ease, and feel relaxed*" showed consistently low correlations with the anxiety subscale, and higher correlations with the depression subscale across a range of patient groups.

Although used extensively with some patient groups (eg. cancer patients) there appears to be relatively little good quality research into the psychometric properties of the HADS for use in a rehabilitation context. One exception is a study conducted by Harter et al. [18] in a number of in-patient rehabilitation clinics in Germany, which assessed the HADS (as compared with the General Health Questionnaire: GHQ) as a screening tool with musculoskeletal patients. The accuracy of the diagnostic ability of the HADS (sensitivity and specificity) compared to standardised interviews was found to be superior to that of the commonly used GHQ. Harter et al. [18], recommend the HADS 'as an efficient instrument to identify patients with musculoskeletal disease and potential psychiatric comorbidity' (p. 743). Based on the findings of their study they propose that the assessment and identification of patients with high levels of anxiety and depression should be given high priority, particularly given their influence on aspects such as quality of life, pain experiences and treatment conformity.

Although the Harter et al. [18] study appears to support the use of the HADS in rehabilitation settings, the authors chose to use the HADS as a total score, rather than as two subscales, as recommended by the scale developers. No justification was given for this decision, and no analyses were conducted to evaluate the appropriateness of this approach. Ideally factor analysis could have been undertaken to assess the structure of the HADS in the rehabilitation context, and to evaluate its psychometric properties.

The aim of this study therefore was to assess the suitability of the HADS as a screening tool for routine use in rehabilitation settings for patients with musculoskeletal disorders. This paper assesses the levels of anxiety and depression in a sample of musculoskeletal patients, and explores the dimensionality of the scale using both exploratory and confirmatory factor analysis.

## Methods

### Participants

The sample consisted of 296 outpatients attending a 6-week musculoskeletal rehabilitation program at Cedar Court HealthSouth Hospital, a private rehabilitation hospital in Melbourne, Australia. There were 152 (51.4%) females, 140 (47.9%) males), 4 cases (1.4 %) sex unspecified. Patients ranged in age from 16 yrs to 80 yrs (mean = 44.3, SD = 12.47). Fifty-five percent of patients reported pain in the lower back, 20% in an upper or lower limb, 15% in the cervical region and 10% in other locations.

### Procedure

The HADS is routinely administered to all patients on admission to the Cedar Court HealthSouth Hospital rehabilitation program as part of standard clinical procedures. Scores on the HADS from all patients attending the program between 2001 and 2003 were extracted from the medical records for this study, with permission from the Hospital Research Committee.

From the original sample of 296 cases, the dataset was randomly divided into two separate and independent sub-samples. The first sample contained 142 cases and was subjected to exploratory factor analysis. The second sample of 154 was used as an independent, holdout sample for confirmatory factor analysis. Although this procedure resulted in two smaller samples it was considered important to use both an exploratory and confirmatory approach with this sample of musculoskeletal patients. It was not appropriate to only use CFA, as the structure of the HADS in musculoskeletal patients had not been previously established using exploratory approaches. Although the sample sizes were smaller than desirable for these analyses, they are similar to other recent studies assessing the HADS on specific clinical samples. Jomeen & Martin [19] conducted both EFA and CFA on a sample of 101 antenatal women; Bedford, Pauw and Grant [20] used a sample of 132 adult psychiatric outpatients; and the sample used by Martin, Tweed & Metcalfe [11] consisted on 160 end stage renal patients. According to Tabachnick and Fidell [21] sample sizes of 150 should be sufficient when solutions show several high loading marker variables (>.80).

### Measures

Hospital Anxiety and Depression Scale (HADS [7]) is a 14-item scale designed to detect anxiety and depression, independent of somatic symptoms. It consists of two 7-item subscales measuring depression and anxiety. A 4-point response scale (from 0 representing absence of symptoms, to 3 representing maximum symptomatology) is used, with possible scores for each subscale ranging from 0 to 21. Higher scores indicate higher levels of disorder. A number of clinical classification schemes have been used to categorise scores on the HADS. In the original article the following cut offs were suggested: 0–7 = 'non-cases'; 8–10 = 'possible case'; 11–21 = 'probable case'.

### Statistical analysis

The reliability of the two subscales was assessed using Cronbach alpha coefficients. The underlying structure of the HADS was explored using both exploratory factor analysis (EFA) and confirmatory factor analysis (CFA). EFA was performed on the first sample using SPSS version 11.5, after first confirming that the data was suitable for factor analysis. Principal components analysis (PCA) was used to extract the factors followed by oblique rotation of factors using Oblimin rotation (delta = 0). The number of factors to be retained was guided by three decision rules: Kaiser's criterion (eigenvalues above 1), inspection of the screeplot, and by the use of Horn's parallel analysis [22] (Horn, 1965). Parallel analysis is one of the most accurate approaches to estimating the number of components [23,24]. The size of eigenvalues obtained from PCA are compared with those obtained from a randomly generated data set of the same size. Only factors with eigenvalues exceeding the values obtained from the corresponding random data set are retained for further investigation. Parallel analysis was conducted using the software developed by Watkins [25].

Confirmatory factor analysis (CFA) using maximum likelihood estimation was conducted on the second holdout sample using AMOS Version 4 [26] to evaluate model fit. Although good model fit can be indicated by a non-significant chi-square, in practice other factors can influence this figure, and therefore a range of fit statistics were assessed. For the incremental fit statistics (Goodness of Fit Index :GFI; the Tucker-Lewis Index :TLI; and the Comparative Fit index: CFI) values less than .90 indicate lack of fit, values between .90 and .95 indicate reasonable fit and values between .95 and 1.00 indicate good fit [21]. Byrne [27] describes the Root Mean-Square Error of Approximation (RMSEA) as the most informative statistic in determining model fit as it takes into account the number of variables that are estimated in the model. RMSEA values are required to be .05 or lower to indicate good fit. Values between .05 and .08 indicate reasonable fit.

## Results

### Descriptive statistics

Scores on the HADS Anxiety scale ranged from 0 to 21, with a mean of 9.26 (SD = 4.43) and a median of 9.0. HADS Depression scale scores also covered the full range from 0 to 21, with a mean of 8.14 (SD = 4.43) and a median of 8.0. Scores on both subscales showed an approximately normal distribution.

The mean scores obtained in this sample on the HADS Anxiety and Depression scales were compared with the scores reported in the literature for other patient groups (see Table 1). The comparison of these subscale scores indicated that this musculoskeletal sample's anxiety and depression levels are higher than each of the clinical samples (cancer [28], end-stage renal disease [29], chronic obstructive pulmonary disease [30], coronary heart disease [31]) samples listed, but lower than that obtained from a sample of psychiatric patients.

**Table 1:** Comparison of HADS anxiety and depression subscale mean scores with other samples

**Sample**	**HADS Anxiety**	**HADS Depression**
Current sample of Musculoskeletal patients	9.26	8.14
Non clinical UK normative sample [37]	6.14	3.68
Coronary heart disease patients [31]	6.14	5.41
End-stage renal patients [29]	6.90	5.20
Breast cancer patients [28]	7.43	3.25
Chronic Obstructive Pulmonary Disease rehabilitation patients [30]	7.10	5.80
Psychiatric patients [20]	13.90	9.90

Patients' scores were classified into the clinical categories defined by the scale's authors. For the anxiety subscale 38.2% of patients were classified as a probable case (score 11 to 21), with a further 23% classified as a possible case (score 8 to 10). On the depression subscale 30.1% were classified as probable cases (score 11 to 21) and 21.3% as possible cases (score 8 to 10).

### Reliability

The Cronbach alpha value for the anxiety subscale was .83, while the Cronbach alpha value for the depression subscale was .84. Both values exceeded the recommended value of .7 [32], indicating adequate internal consistency.

### Exploratory factor analysis

The sample was first assessed for its suitability for factor analysis. Bartlett's Test of Sphericity was highly significant (p < .001) and the Kaiser-Meyer-Olkin (KMO) measure of sampling adequacy value of .9 supported the factorability of the matrix [21]. Principal Components Analysis (PCA) revealed two eigenvalues exceeding 1, explaining 41.4% and 11.4% of the variance respectively. Only these first two factors exceeded the criterion value obtained from Parallel Analysis [25]. Inspection of the screeplot also supported a two factor solution. Following Oblimin rotation the two factors showed a moderate intercorrelation (r = .48). Inspection of the pattern matrix (Table 2) showed a relatively clear two-factor solution in line with Zigmond & Snaith's [7] anxiety and depression factors, with the exception of Item 7. This anxiety item loaded strongly (.64) and inappropriately onto the depression factor, and barely loaded (.005) on the anxiety factor. Two other anxiety items also showed crossloadings on the depression factor (item 1 = .378; item 5 = .274), however both of these items showed higher relative loadings on the Anxiety factor (item 1 = .434 and item 5 = .531).

**Table 2:** Pattern and structure matrix for PCA with oblimin rotation of two factor solution

**HADS Item**	**Depression**		**Anxiety**		**Communalities**
	Pattern	Structure	Pattern	Structure	
HADS12-dep	**.868**	**.820**	-.101	.312	.681
HADS2-dep	**.785**	**.713**	-.151	.222	.526
HADS8-dep	**.709**	**.675**	-.071	.265	.460
HADS4-dep	**.669**	**.753**	.177	.494	.591
HADS7-anx	**.641**	**.643**	.005	.309	.414
HADS14-dep	**.572**	**.630**	.124	.395	.409
HADS6-dep	**.552**	**.693**	.295	.557	.547
HADS10-dep	**.537**	**.618**	.173	.427	.406
HADS9-anx	-.098	.311	**.863**	**.816**	.674
HADS13-anx	-.010	.395	**.852**	**.848**	.718
HADS3-anx	-.016	.368	**.807**	**.800**	.640
HADS11-anx	.030	.299	**.567**	**.581**	.338
HADS5-anx	.274	.527	**.531**	**.662**	.496
HADS1-anx	.378	.584	**.434**	**.614**	.487

Note: Bolded items indicate major loadings for each item.

Analysis of the structure matrix indicated good discrimination between the factors. For the depression component, the lowest factor loading for depression items was .62 for Item 10, which was still higher than the highest loading (Item 1, loading at .58) on the depression factor of an anxiety item (except for item 7). The anxiety component also showed good discrimination: the lowest loading anxiety item (Item 11, loading at .58) was still higher than the highest loading depression item onto the anxiety component (Item 6, loading at .56).

Overall these results support the bi-dimensionality of the HADS, however the content of the factors obtained do not fully support the original scale structure proposed by the authors. The major inconsistency in relation to this sample is the tendency of one of the anxiety items (item 7: "*I can sit at ease and feel relaxed'*) to show more substantial loadings on the Depression factor. It was therefore decided to explore the structure of the HADS with this item removed.

PCA with oblimin rotation was repeated with item 7 removed. This resulted in a 13-item scale (HADS-13), with six anxiety items and seven depression items. The pattern matrix (Table 3) showed a separation of the anxiety and depression subscales. All items loaded above .45 on their respective factors; however Anxiety items 1 and 5 also showed some loading on the Depression factor (item 1 = .339, item 5 = .270). The factors correlated substantially (r = .47). The anxiety scale (without item 7) had a Cronbach alpha value of .84, indicating good internal consistency.

**Table 3:** Pattern and Structure Matrix for Principal Components Analysis of HADS-13

**HADS Item**	**Depression**		**Anxiety**		**Communalities**
	Pattern	Structure	Pattern	Structure	
HADS 12-dep	**.871**	**.827**	.092	-.318	.691
HADS 2-dep	**.816**	**.740**	.162	-.222	.568
HADS 4-dep	**.695**	**.775**	-.169	-.496	.622
HADS 8-dep	**.688**	**.666**	.048	-.276	.445
HADS 10-dep	**.586**	**.653**	-.142	-.418	.442
HADS 14-dep	**.580**	**.638**	-.124	-.397	.419
HADS 6-dep	**.506**	**.663**	-.333	-.571	.525
HADS 13-anx	-.019	.383	**-.855**	**-.846**	.716
HADS 9-anx	-.081	.318	**-.846**	**-.808**	.659
HADS 3-anx	-.009	.368	**-.800**	**-.795**	.633
HADS 11-anx	-.012	.269	**-.596**	**-.590**	.348
HADS 5-anx	.270	.523	**-.538**	**-.665**	.499
HADS 1-anx	.339	.559	**-.467**	**-.626**	.482

Note: Bolded items indicate major loadings for each item.

The HADS-13 (with item 7 removed) showed simple structure consistent with Zigmond and Snaith's [7] original conception of the factor structure for the HADS. Unlike the 14-item scale, all the items loaded onto the correct subscales.

### Confirmatory factor analysis

**Figure 1:**
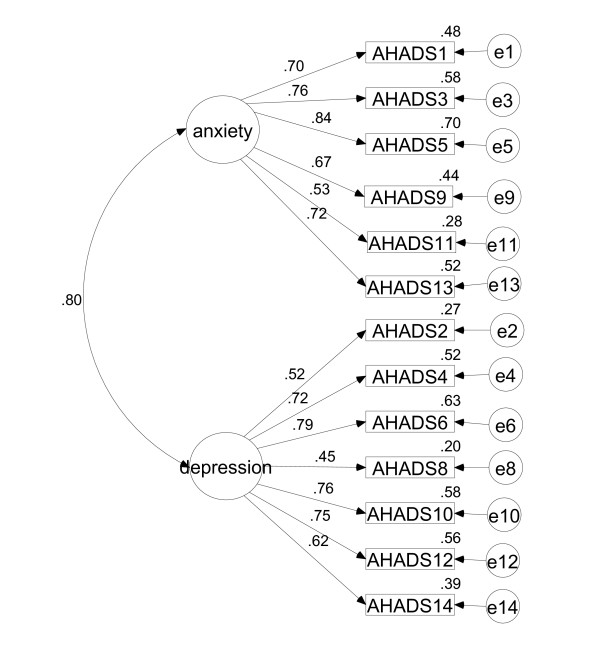
HADS-13: Two-factor confirmatory factor model.

Confirmatory factor analysis using maximum likelihood estimation was conducted on the second independent sample of 154 cases. A number of alternative models were investigated. A 13-item, two-factor model, as identified in the exploratory factor analysis, was investigated allowing the factors to freely correlate (Figure 1). Factor loadings in this model were statistically significant. Although the chi-square test was significant [chi square (64) = 96.56, p = .005], the other fit indices indicated good fit. The GFI statistic (.916) was reasonable, and the TLI (.953), CFI (.961), and RMSEA (.058) indicated good fit. Inspection of the residuals and modification indices revealed no items failing to correlate or other required paths.

Whilst the structure matrix in the exploratory factor analysis indicated that the components were clearly distinguishable constructs, the structure matrix for this CFA model indicated that the items were not distinct from each other. On the anxiety subscale, the lowest loading for anxiety items was .53, whilst the highest loading of depression items onto the anxiety subscale was .63. This was reflected in the depression subscale, where the lowest loading of depression items was .45, whilst the highest loading of anxiety items onto the depression subscale was .67. This indicates a large amount of overlap between the factors, which is also supported by the strong correlation (.80) between the two factors.

Given evidence of the strong overlap of the two factors it was decided to formally test a one-factor model of generalised psychological distress, as proposed by previous researchers [10,11]. Two models were tested: the full 14 item original version of the HADS and the 13 item version with item 7 removed.

**Figure 2:**
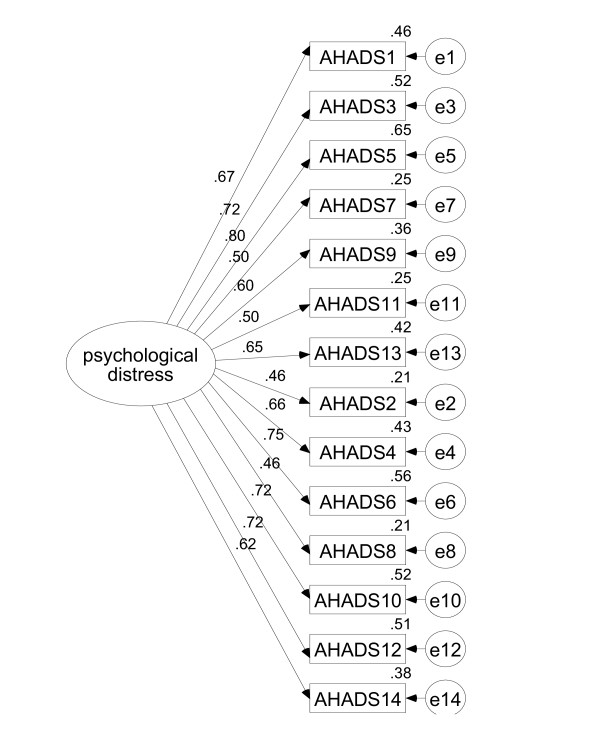
One factor, 14 item confirmatory factor model.

Fit statistics for the one-factor model for all 14 items (displayed in Figure 2) showed poor fit to the data (see Table 4). The chi-square statistic was significant [chi square (77) = 190.19, p < .001). The GFI (.826), TLI (.852) and CLI (.875) fit statistics were all lower than the recommended guidelines, indicating misfit. The RMSEA (.098) was barely adequate. A one-factor model for the 13 item version also showed poor model fit (see Table 4).

**Table 4:** Comparative fit indices for five CFA HADS models

	GFI	TLI	CFI	RMSEA
HADS 13 items 2 factors	.916	.953	.961	.058
HADS 14 items 2 factors	.884	.919	.932	.073
HADS 14 items 1 factor	.826	.852	.875	.098
HADS 13 items 1 factor	.843	.869	.891	.096
HADS 14 item 3 factor (Dunbar et al. final model [12])	.907	.943	.955	.060

Although not supported by the results of the EFA analyses, a three factor modified model proposed by Dunbar [12] (based on Clark and Watson's tripartite theory) was also tested with this sample (see Dunbar p. 88 for specific details of the model). The model fit statistics shown in Table 4 were adequate, but not as good as those obtained from the 13-item 2 factor solution.

Overall the best fitting model for this data was a two correlated factor model representing anxiety and depression, but with item 7 removed from the scale.

## Discussion

The results of this study revealed high levels of anxiety and depression among patients undergoing rehabilitation for musculoskeletal disorders. The mean score obtained on each of the HADS subscales was higher than other reported samples of patients with breast cancer [33], renal disease [29], chronic obstructive pulmonary disease [30] and coronary heart disease [31], but lower than for psychiatric patients [20]. In the current sample more than 60% of patients were defined as 'cases' or 'possible cases' of anxiety, and more than 50% of patients were defined as 'cases' or 'possible cases' of depression.

Previous reviews of prevalence rates [34] suggest that patients with chronic pain (including fibromyalgia, and back pain) had higher prevalence of anxiety and depression than patients with oncology, cardiac and neurological disorders. Similar high rates for anxiety were reported in a study of breast cancer patients [28], with a total of 68.2% of patients being classified as 'possible' (46.4%) or 'probable' (21.8%) cases. For this sample, however, only 12.7% of the cancer patients were classified as 'possible' or 'probable' cases on the depression subscale. In a recent study of cardiac patients [31] 40% of patients were classified as a 'possible' or 'probable' case on the depression scale. Compared to other samples, this current sample of musculoskeletal patients had substantial levels of both anxiety and depression.

These results suggest that issues concerning psychological comorbidity with chronic pain need to be addressed, particularly in patients with musculoskeletal disease. Both anxiety and depression can have a negative impact on patients' quality of life, on their perception and response to pain, their treatment adherence and on their ability to benefit from treatment programs [18]. Screening procedures to detect patients with elevated anxiety and depression are important in rehabilitation settings to allow the identification of patients requiring additional psychological assessment, prior to commencement of programs. The results of this study suggest that, with one minor adjustment (removal of item 7), the HADS may be suitable for use as a screening instrument for use in rehabilitation settings with musculoskeletal patients.

As detailed in the introduction, a number of concerns have been raised in recent years concerning the dimensionality of the HADS and the behaviour of individual items. The results of the current study generally provide support for the bi-dimensionality of the HADS, however the content of the factors obtained do not fully concur with the original scale conceptualisation. The major inconsistency in relation to this sample is the tendency for item 7 ("*I can sit at ease and feel relaxed'*) to show more substantial loadings on the Depression factor, rather than its Anxiety factor. Problems with this item have been reported by a number of other researchers working with different clinical patient groups [20,31,34-36]. Dunbar et al. [12], in their study of the HADS, adjusted the CFA model to allow item 7 to also load on the depression factor. It was suggested that this crossloading may be due to the fact that the item taps the loss of a pleasurable state (that is, 'being at ease'), consistent with the other depression items tapping anhedonia (loss of pleasure).

In further considering the content of this item it is not surprising that this item may behave 'inappropriately', given the nature of the musculoskeletal disorders experienced by patients in this sample. For many patients with back injuries and chronic pain, sitting is not always comfortable and many would not 'feel at ease'. This is not necessarily due to a psychological disturbance (anxiety), but instead is likely to be due to physical factors.

Given the clearly inappropriate loadings noted in EFA it was decided to remove item 7 from the scale in the subsequent CFA analyses. We did not feel there were sufficient theoretical, conceptual or clinical grounds to support the inclusion of item 7 with the depression items. It was considered inappropriate to rely solely on statistical evidence in this study to reassign the item, particularly given its inconsistent behaviour reported in the literature. By not including item 7 with the depression items, the Depression subscale remains consistent with that recommended by the original authors, and in the format used by the majority of previous researchers and clinicians.

The 13-item two-factor CFA (excluding item 7) indicated that the revised model was a good fit to the data, with 7 items representing depression and 6 items representing anxiety. A one-factor general psychological distress model was also examined to test proposals put forward by some authors concerning the use of the HADS as a unidimensional scale [10,11]. The one factor model however, did not display good fit to the data.

The two-factor solution obtained in this study contributes to the growing body of papers investigating the factor structure of the HADS, as reviewed by Bjelland et al. [8]. The consistent correlation between the two subscales in this study (r = .48) indicates that the overlap between the two factors could be inherent in the nature of these dimensions. Roberts et al. [9] describe this as a "somewhat 'muddy' area in the middle where symptoms common to both syndromes overlap" (p 380).

In this study Clark and Watson's [16] tripartite model was not supported by EFA but was tested for comparative purposes using CFA. The three factor model specified by Dunbar et al. [12] was tested, and although it showed adequate model fit, the fit statistics were not as good as those recorded for the 13-item two factor model with item 7 removed. It is questionable whether the HADS would be an appropriate place to fully test a tripartite model, due to the small item pool, the emphasis of anhedonia items in the depression subscale, and the lack of somatic items. The clinical utility of a three subscale structure of the HADS is also questionable, an issue raised by Rodgers and colleagues [28]. The separation of negative affectivity, autonomic anxiety and anhedonic depression, of interest theoretically, is of little use to clinicians wishing to use the HADS to quickly and simply screen their patients for elevated levels of anxiety and depression.

The results of this current study suggest that modifications to the original structure of the HADS [7] are necessary when using the scale in a sample of musculoskeletal patients. Item 7 should not be included in the calculation of the HADS Anxiety subscale scores. The Depression subscale however remains consistent with the original scale design and can be used with the cut points recommended by the scale authors [7]. Further research will be necessary to establish new cut-points for the revised 6-item anxiety subscale by validating it against a structured clinical interview.

## Conclusion

The high levels of anxiety and depression detected in the patients in this study suggest that screening for psychological comorbidity is important in musculoskeletal rehabilitation settings. It is necessary, however, for clinicians, who are considering using the HADS as a screening tool, to first assess its suitability with their particular patient group. Although EFA and CFA supported the presence of two subscales representing anxiety and depression, the results with this musculoskeletal sample suggest that item 7 should be removed from the anxiety subscale.

## Authors' contributions

JP designed the study, obtained the data, and supervised the statistical analyses conducted. CB undertook the literature review and performed the statistical analyses. Both authors contributed to the preparation of the article. Both authors read and approved the final manuscript.
